# Supplementary material for: Quantifying the Effect of Supplementation with Algae and Its Extracts on Glycolipid Metabolism: A Meta-Analysis of Randomized Controlled Trials
Source: Nutrients. 2020 Jun 8;12(6):1712. doi: 10.3390/nu12061712 (PMC7352414; doi:10.3390/nu12061712)
Supplement: Supplementary file 1 [file nutrients-12-01712-s001.pdf]

Supplementary materials

A

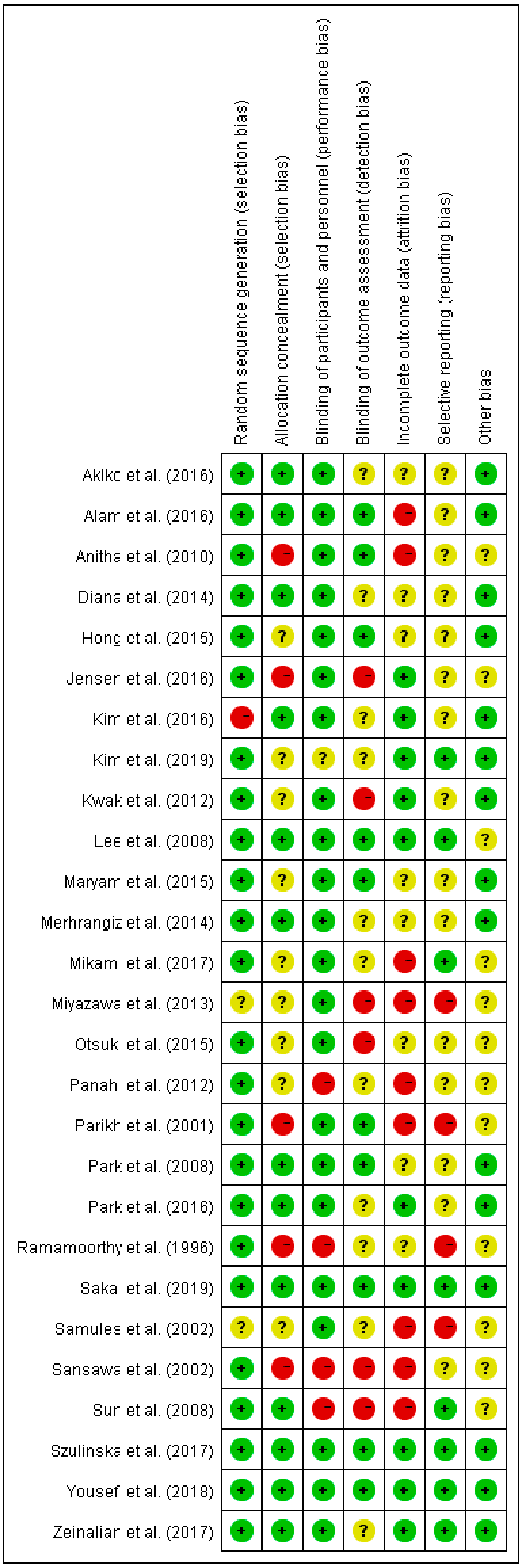

B

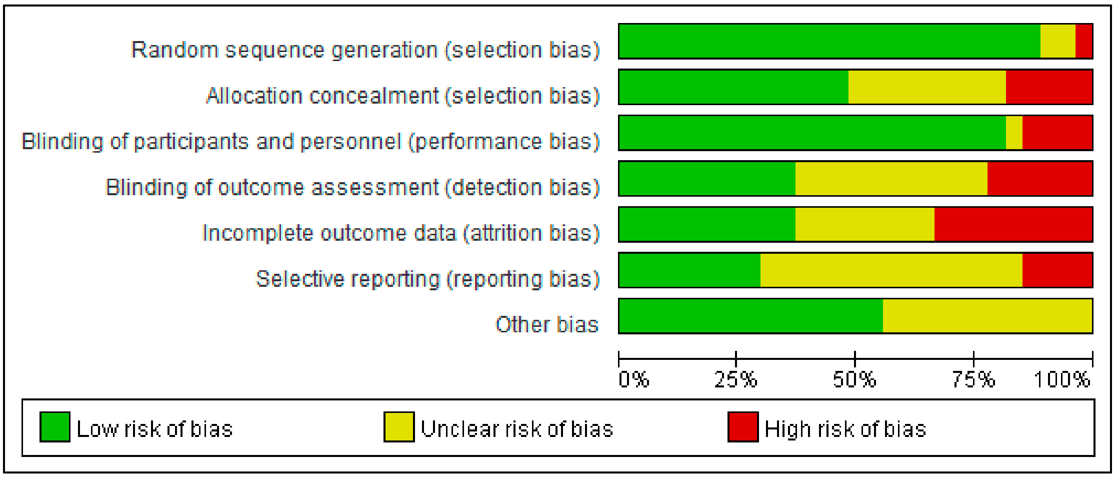

Figure S1. Quality assessment of the included studies. labeling an item as a "question mark" indicated an unclear or unknown risk of bias; labeling an item as a "negative sign" indicated a high risk of bias; labeling an item as a "positive sign" indicated a low risk of bias.

A

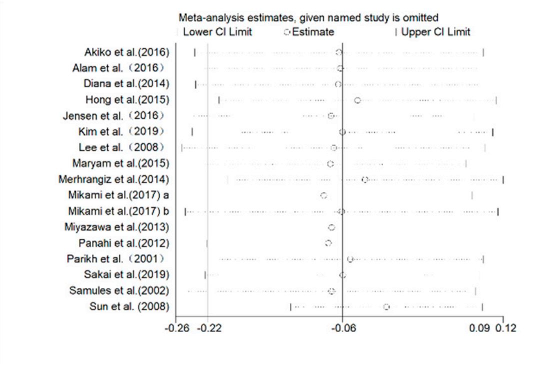

B

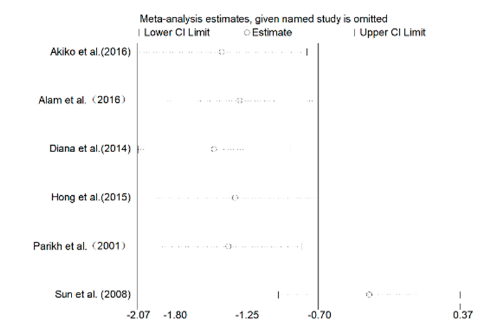

C

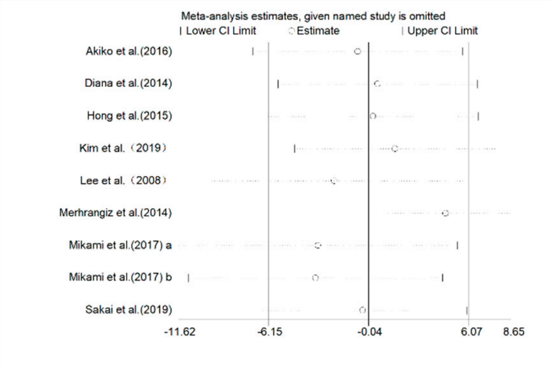

D

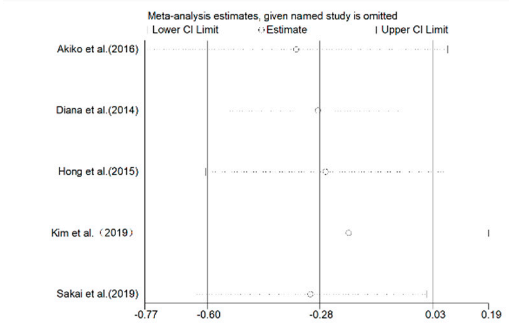

E

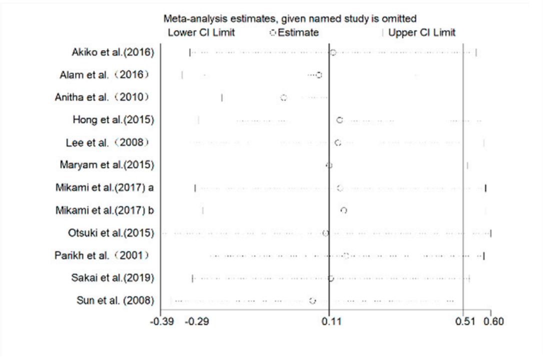

F

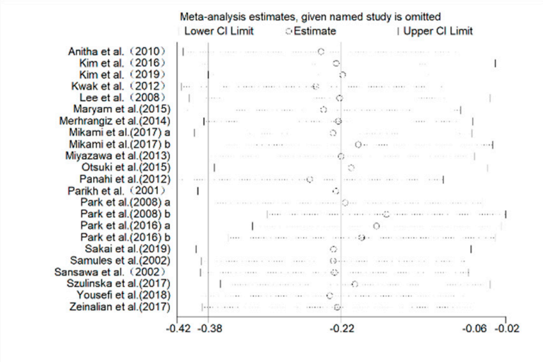

G

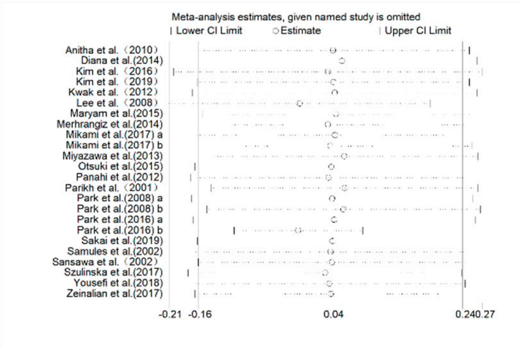

H

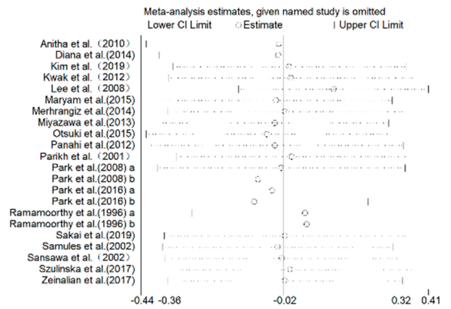

I

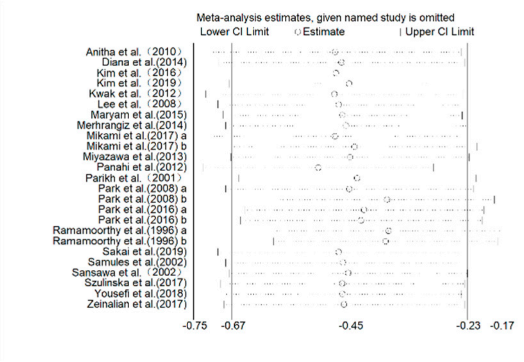

Figure S2. Sensitivity analysis was conducted on outcomes of FPG (A), 2hPBG (B), insulin (C), HOMA-IR (D), HbA1c (E), HDL-C (F), LDL-C (G), TG (H) and TC (I) using the leave-one-out method.

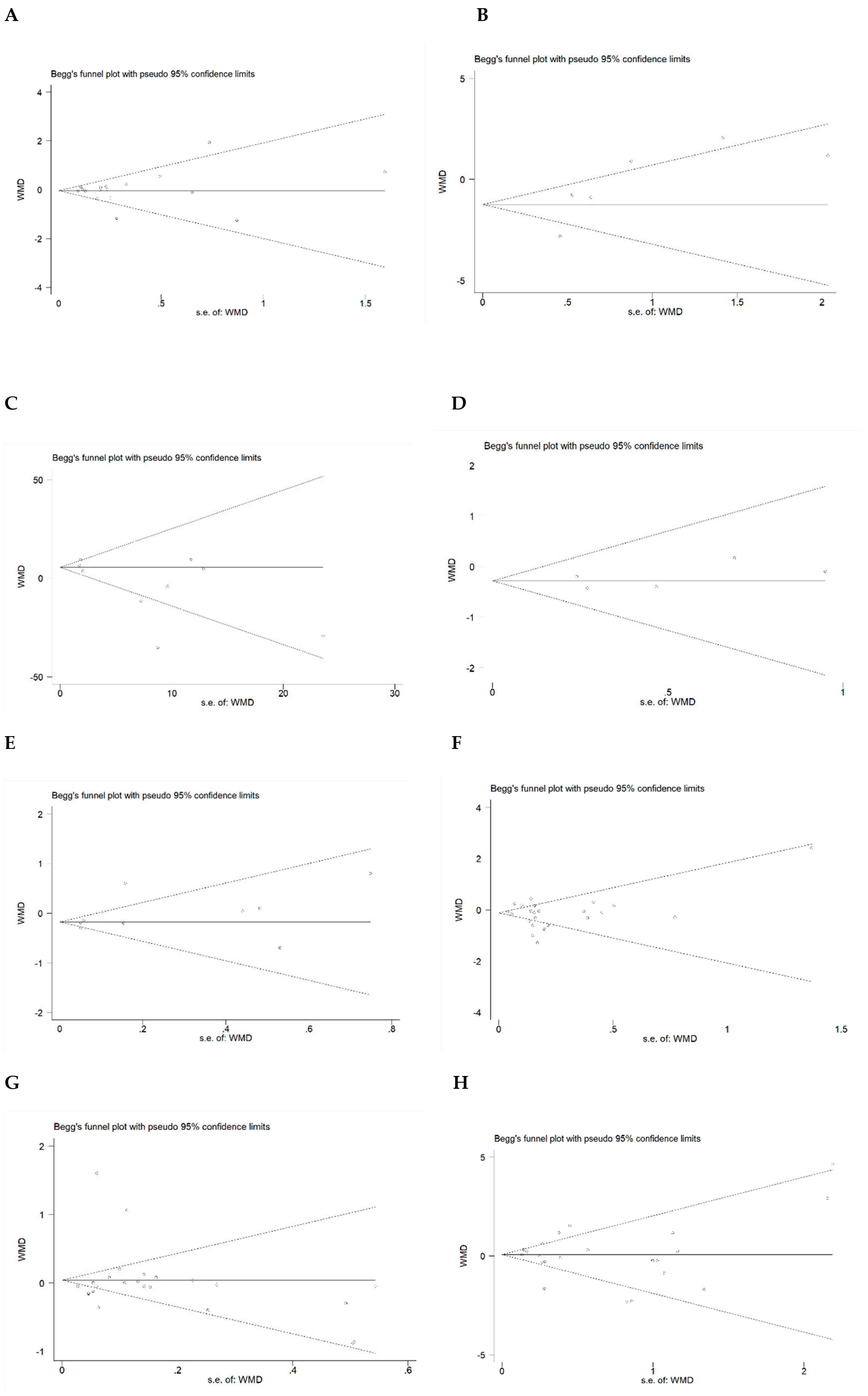
A
Begg's funnel plot with pseudo 95% confidence limits
WMD
s.e. of: WMD
B
Begg's funnel plot with pseudo 95% confidence limits
WMD
s.e. of: WMD
C
Begg's funnel plot with pseudo 95% confidence limits
WMD
s.e. of: WMD
D
Begg's funnel plot with pseudo 95% confidence limits
WMD
s.e. of: WMD
E
Begg's funnel plot with pseudo 95% confidence limits
WMD
s.e. of: WMD
F
Begg's funnel plot with pseudo 95% confidence limits
WMD
s.e. of: WMD
G
Begg's funnel plot with pseudo 95% confidence limits
WMD
s.e. of: WMD
H
Begg's funnel plot with pseudo 95% confidence limits
WMD
s.e. of: WMD

I

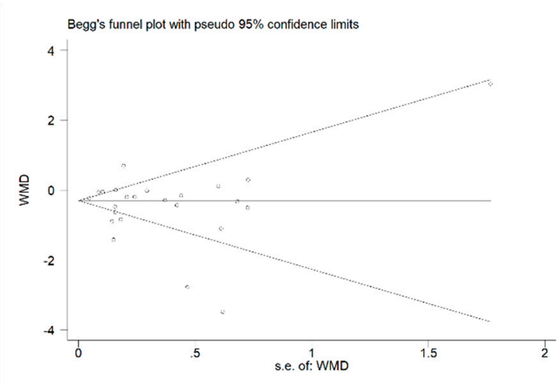

Figure S3. Funnel plots with pseudo 95% CIs for publication bias of FPG (A), 2hPBG (B), insulin (C), HOMA-IR (D), HbA1c (E), HDL-C (F), LDL-C (G), TG (H) and TC (I).
